# Identification and Replication of Urine Metabolites Associated With Short-Term and Habitual Intake of Sweet and Fatty Snacks in European Children and Adolescents

**DOI:** 10.1016/j.tjnut.2024.09.026

**Published:** 2024-09-25

**Authors:** Jantje Goerdten, Samuel Muli, Jodi Rattner, Mira Merdas, David Achaintre, Li Yuan, Stefaan De Henauw, Ronja Foraita, Monica Hunsberger, Inge Huybrechts, Lauren Lissner, Dénes Molnár, Luis A Moreno, Paola Russo, Toomas Veidebaum, Krasimira Aleksandrova, Ute Nöthlings, Kolade Oluwagbemigun, Pekka Keski-Rahkonen, Anna Floegel

**Affiliations:** 1 Leibniz Institute for Prevention Research and Epidemiology (BIPS), Bremen, Germany; 2 Unit of Nutritional Epidemiology, Department of Nutrition and Food Sciences, University of Bonn, Bonn, Germany; 3 International Agency for Research on Cancer (IARC), Lyon, France; 4 Department of Public Health and Primary Care, Ghent University, Ghent, Belgium; 5 School of Public Health and Community Medicine, Institute of Medicine, Sahlgrenska Academy, University of Gothenburg, Gothenburg, Sweden; 6 Department of Pediatrics, Medical School, University of Pécs, Pécs, Hungary; 7 GENUD (Growth, Exercise, NUtrition and Development) Research Group, Faculty of Health Sciences, University of Zaragoza, Instituto Agroalimentario de Aragón (IA2) and Instituto de Investigación Sanitaria Aragón (IIS Aragón), Zaragoza, Spain; 8 Consorcio CIBER, M.P. Fisiopatología de la Obesidad y Nutrición (CIBERObn), Instituto de Salud Carlos III (ISCIII), Madrid, Spain; 9 Institute of Food Sciences, CNR, Avellino, Italy; 10 National Institute for Health Development, Tallinn, Estonia; 11 Faculty of Human and Health Sciences, University of Bremen, Bremen, Germany; 12 Section of Dietetics, Faculty of Agriculture and Food Sciences, Hochschule Neubrandenburg—University of Applied Sciences, Neubrandenburg, Germany

**Keywords:** biomarker identification, metabolite biomarkers of food intake, untargeted metabolomics, sweet and fatty snacks, children and adolescents

## Abstract

**Background:**

Intake of sweet and fatty snacks may partly contribute to the occurrence of obesity and other health conditions in childhood. Traditional dietary assessment methods may be limited in accurately assessing the intake of sweet and fatty snacks in children. Metabolite biomarkers may aid the objective assessment of children’s food intake and support establishing diet–disease relationships.

**Objectives:**

The present study aimed to identify biomarkers of sweet and fatty snack intake in 2 independent cohorts of European children.

**Methods:**

We used data from the IDEFICS/I.Family cohort from baseline (2007/2008) and 2 follow-up examination waves (2009/2010 and 2013/2014). In total, 1788 urine samples from 599 children were analyzed for untargeted metabolomics using high-resolution liquid chromatography-mass spectrometry. Short-term dietary intake was assessed by 24-h dietary recalls, and habitual dietary intake was calculated with the National Cancer Institute method. Data from the Dortmund Nutritional and Anthropometric Longitudinal Designed (DONALD) cohort of 24-h urine samples (*n* = 567) and 3-d weighted dietary records were used for external replication of results. Multivariate modeling with unbiased variable selection in R algorithms and linear mixed models were used to identify novel biomarkers. Metabolite features significantly associated with dietary intake were then annotated.

**Results:**

In total, 66 metabolites were discovered and found to be statistically significant for chocolate candy; cakes, puddings, and cookies; candy and sweets; ice cream; and crisps. Most of the features (*n* = 62) could not be annotated. Short-term and habitual chocolate intake were positively associated with theobromine, xanthosine, and cyclo(L-prolyl-L-valyl). These results were replicated in the DONALD cohort. Short-term candy and sweet intake was negatively associated with octenoylcarnitine.

**Conclusions:**

Of the potential metabolite biomarkers of sweet and fatty snacks in children, 3 biomarkers of chocolate intake, namely theobromine, xanthosine, and cyclo(L-prolyl-L-valyl), are externally replicated. However, these potential biomarkers require further validation in children.

## Introduction

In epidemiologic studies, dietary intake is commonly assessed through self-reports using instruments such as food frequency questionnaires (FFQs), 24-h dietary recalls (24-HDRs), and 3-d weighted dietary records (3d-WDRs) [1]. These dietary assessment methods present some challenges, such as the potential for misreporting [2]. Although progress has been made in generating evidence for diet–disease relationships in adults, evidence is lacking for children and adolescents [3]. This might be partially due to the additional difficulties in dietary intake assessment for children and adolescents, namely, unstructured eating patterns, concerns with self-image, or problems in conceptualizing time, to mention only a few [4]. Hence, objective measures of nutrient and dietary intake are needed to aid or even replace the traditional assessment methods. Indeed, dietary biomarkers, such as urinary nitrogen [5] or vitamin C [6], have been used as objective indicators of dietary intake in dietary validation studies [7,8].

The rise of omics technologies has paved the way for objective measures of dietary intake, also known as biomarkers of food intake (BFI) [1,9,10]. Specifically, metabolomics has emerged as a powerful discovery method for the analysis of biospecimens, such as urine, allowing for the subsequent identification of novel BFIs [11]. In untargeted metabolomics, low molecular weight compounds, called metabolites, are comprehensively analyzed without previous selection of targeted analytes. Metabolites are the end products of metabolism, many of which reflect short-term or habitual food intakes [10]. Furthermore, measuring the food metabolome through untargeted metabolomics can yield a vast number of metabolites [10]. Urine biosamples are a great resource because the collection is noninvasive and can be collected at home by the study participants [12]. Therefore, analyzing the urine-based food metabolome offers a unique opportunity to identify novel BFIs.

Over the last decade, there have been enormous efforts to identify biomarkers of various nutrients, foods, food groups, or dietary patterns [[13], [14], [15], [16], [17], [18], [19], [20], [21], [22], [23]]. However, most of the studies have focused on adult populations, with only a few including children and/or adolescents [13,24,25]. Furthermore, most studies only internally validated their findings and lack external replication [26]. External validation is an important step in increasing the reliability of BFI.

For children and adolescents, the most studied BFIs are for fruit and vegetable intake [25,27]. Additionally, 1 study identified BFIs for meat and fish intake [24]. To date, to our knowledge, there are no candidate BFIs for sweet and fatty snacks in children and adolescents. This gap exists partly because few biomarker discovery studies focus on children and partly because sweet and fatty snack intake is especially difficult to assess [4]. Moreover, only a few candidate BFIs exist for adult populations, mainly for cocoa, liquorice, and potato crisps [17,23].

BFIs for sweet and fatty snacks in children are missing. If identified, these BFIs may provide important insights into understanding childhood diet–disease associations. Hence, this study aimed to identify novel BFIs of short-term and habitual intake of sweet and fatty snack foods in the repeatedly measured food metabolome from 2 independent longitudinal cohorts of European children and adolescents.

## Methods

### IDEFICS/I.Family

The Identification and Prevention of Dietary- and Lifestyle-induced Health Effects in Children and Infants (IDEFICS) and I.Family cohort served as the main cohort for identifying biomarkers [28,29]. Data were gathered in 8 study centers across Europe—Belgium, Cyprus, Estonia, Germany, Hungary, Italy, Spain, and Sweden [29]. The study aimed to determine the etiology of overweight, obesity, and related health outcomes. Thus, repeated measurements of lifestyle, behavior, and medical parameters were taken, at baseline and over 5 follow-up time points. Children were recruited from kindergarten and school settings [28]. The baseline examination, conducted between 2007 and 2008, included >16,000 children aged 2 to10 y [29]. Ethical approval was obtained from the corresponding national or local ethics committees of the participating countries. A more in-depth description of the IDEFICS/I.Family cohort can be found elsewhere [28,29].

**FIGURE 1:**
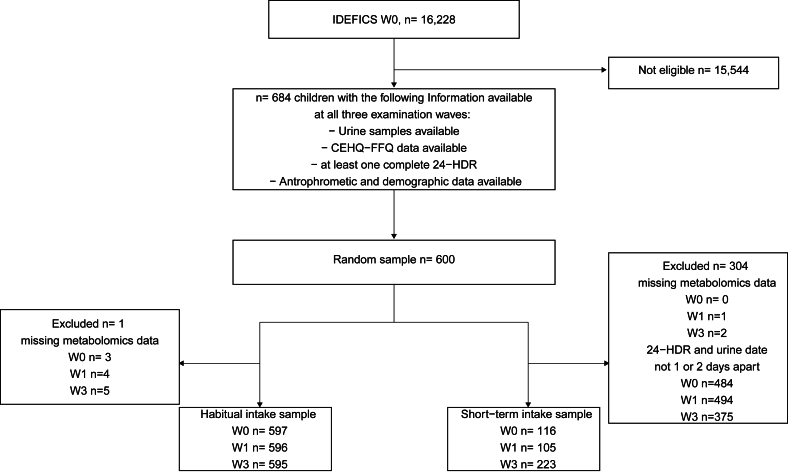
Flow diagram of the study sample selection in the discovery cohort (IDEFICS/I.Family). Abbreviations: CEHQ, Children’s Eating Habits Questionnaire; FFQ, food frequency questionnaire; 24-HDR, 24-h dietary recall.

For this study, a random subsample (*N* = 600) of the original cohort was selected. Sample size calculations were performed a priori based on metabolite measurements at 1 time point. We expected a statistical power of >80%. Eligible participants had available urine samples, dietary, demographic, and anthropometric data at baseline and 2 follow-up time points. Figure 1 shows flow diagram of the study sample selection. Data from 3 time points, including baseline, were used in this study. These time points are referred to as baseline (W1), second examination wave (W2), and third wave (W3) hereafter. W2 data were collected from 2009 to 2010, and data collection for W3 took place between 2013 and 2014.

### Covariate assessment

Anthropometric data from all participants were collected using standardized methods [[28], [29], [30]]. Body weight of the study participants was measured by a trained study nurse using a TANITA 418/420 digital scale [31]. Height was measured using a Seca 225/213 stadiometer [31]. BMI was calculated by dividing the weight in kilograms by the squared height in metres. Age-specific and sex-specific BMI *z*-scores according to Cole and Lobstein [32] were used in the statistical analysis. Demographic data, such as age, sex, and country of residence were routinely collected during baseline and follow-ups [28,29].

### Dietary assessment

The dietary intake of participants was recorded using the 24-HDR and FFQ instruments. For the 24-HDR, a computer-based program, the Self-Administered Children and Infant Nutrition Assessment, was used at W1 and W2, and a web-based program, the Self-Administered Children, Adolescents, and Adult Nutrition Assessment, was used at W3. The FFQ section of the Children’s Eating Habits Questionnaire was applied at each follow-up time point [33,34].

Study participants were asked to complete ≥2 weekday 24-HDRs and 1 on the weekend. If these were not completed, participants were reminded by telephone or e-mail [35]. The 24-HDR recorded the dietary intake (ie, the type and amount of foods and beverages) over the last 24 h [36]. The intake was structured into 6 meal occasions: breakfast (first intake after waking up), mid-morning snack, lunch, afternoon snack, dinner, and evening snack, with the possibility to add more eating occasions if required. Quantities of foods were estimated using standardized photographs of serving sizes, standard portions, customary packaging sizes, and meals in pieces or slices. For the 24-HDR based on the Self-Administered Children and Infant Nutrition Assessment program, a proxy respondent, that is, primary caretaker of the child, recalled the dietary intakes. Otherwise, children or adolescents recalled the dietary intake of the last 24 h themselves, with the help from primary caretakers if needed. A dietician or trained study nurse was present during each 24-HDR to answer any questions [30]. Several studies were undertaken to validate the 24-HDR in the IDEFICS/I.Family cohort [33].

The FFQ comprised 60 food items grouped into 15 food groups: specifically, vegetables, fruit, breakfast cereals, milk, yogurt, cheese, fish, meat and meat products, eggs, meat replacement products and soy products, spreadable products, cereal products and snacks, plant oil, and beverages [37]. Possible answers for the FFQ section were never/less than once a week, 1–3 times a week, 4–6 times a week, 1 time a day, 2 times a day, and 3 times or more a day. The previous month was set as the reference period for the FFQ. During W1 and W2, specifically, the primary caretaker of the child responded on behalf of the child. However, in W3, adolescents aged 12 y and over completed the FFQ section themselves. The measured food consumption of the FFQ was previously validated against nutrients measured in blood and urine [35,38].

### Short-term dietary intake

Short-term dietary intake was defined as any food intake the day or 2 d before the urine collection. To derive the short-term dietary intake of chocolate candy (including cocoa powder used for chocolate beverages); chocolate and nut spread; potato crisps; jelly candy; candy and sweets (nonchocolate); cakes, puddings, and cookies; and ice cream, the dietary intakes of these foods were estimated in grams from the 24-HDR data. If several foods could be classified as the defined food group, the intake amounts were summed up. An overview of the food names used from the 24-HDR to derive the food group intakes is provided in the Supplemental Material. For some children, the difference between urine sample collection and assessed dietary intake was >2 d. These children were excluded from the analysis of short-term food intake. Children for whom no metabolomics data were available owing to laboratory error were also excluded. Thus, the final study sample for short-term dietary intake was as follows: *n* = 444—for W1: *n* = 116, W2: *n* = 105, and W3: *n* = 223. There were 45 participants with 3 repeated measurements, 58 had 2, and 193 had 1 measurement.

We performed 2 sensitivity analyses. In the first, the short-term intake sample was limited to study participants with urine samples collected on the same day of the 24-HDR. In the second, we included coffee and tea intake as a further covariate in the statistical model to investigate any changes in the results for chocolate candy intake. Coffee and tea intake was derived from the 24-HDR (Supplemental Material).

### Habitual dietary intake

Habitual dietary intake was defined as the estimated usual dietary intake per day for each participant. The method developed by the United States National Cancer Institute (NCI) was used to estimate individual habitual dietary intake (grams per day) [39]. To calculate habitual dietary intake for the study population the food groups defined from the 24-HDR and the dietary frequency questions from the FFQ were grouped (Supplemental Material) and analyzed according to Kipnis et al. [40]. 24-HDRs with an estimated total energy intake below 500 kcal were excluded from the analysis. The computation of the habitual dietary intake was stratified by sex and adjusted for age and BMI *z*-score. The habitual dietary intake was calculated for chocolate candy (including cocoa powder); savory and fatty snacks; candy and sweets (nonchocolate); crisps (potato and other); cakes, puddings and cookies; and ice cream. Metabolomics data were unavailable for very few children owing to laboratory errors. Thus, the final study sample consisted of *n* = 1788—for W1, *n* = 597; for W2, *n* = 596; and for W3, *n* = 595, respectively. All children who were included in the study sample for short-term dietary intake were also included in the study sample for habitual dietary intake.

### Urine sample collection

Sample collection for the IDEFICS/I.Family cohort followed a standardized procedure across all participating study centers [41]. The morning urine was collected at home by the primary caregivers directly after waking. The caregivers received a urine collection kit and an instruction sheet [41]. Caregivers were asked to cool the urine sample in the refrigerator if the time until arrival at the study center was longer than 2 h. At the study center, the urine samples were cooled to −20 °C. At regular intervals, the biosamples were shipped on dry ice to the central biorepository, where they were frozen at −20 °C and stored [35,42].

### Dortmund Nutritional and Anthropometric Longitudinal Designed

For the external replication of the identified metabolites the Dortmund Nutritional and Anthropometric Longitudinal Designed (DONALD) cohort was used [43]. The DONALD cohort is an open cohort study with 2375 participants recruited between 1985 and 2022 [44]. The participants were followed from infancy (3 mo of age) to adulthood at regular intervals during which dietary, health, and developmental data were collected. Dietary data were collected with 3d-WDR at each examination interval. Parents or children were asked to weigh their food and fill in the dietary records. Dieticians checked the records for plausibility. 24-h urine samples were collected at every interval from age 3 y onward. The urine samples were collected by the parent at home on the third day of the 3d-WDR. The biological samples were stored at the DONALD study center. Details of the DONALD cohort are available elsewhere [43,44].

For this project, a random sample of 300 children with ≥2 available urine samples and 2 3d-WDR were selected. In total, 600 urine samples were available. The final DONALD replication sample included 297 participants, of whom 270 had repeated measurements and 27 had single measurements available. The reduction in participants was due to an incomplete 3d-WDR and/or a mismatch between the 3d-WDR date and the urine collection date. This resulted in 567 measurements in the replication cohort.

### Sample preparation and randomization

For this study, 1800 urine samples from the IDEFICS/I.Family cohort and 600 urine samples from the DONALD cohort were initially shipped from Bremen and Bonn, both in Germany, to the laboratory at the International Agency for Research on Cancer (IARC) for metabolomics analyses. The study samples were anonymized and randomized before shipment. The repeated samples for each study participant were analyzed next to each other in random order, and sample pairs were randomized across the batch. Randomization of the samples was further stratified by country for the multicenter IDEFICS/I.Family cohort samples, to ensure an equal proportion of samples from each country on each plate. Quality control samples were prepared from a sample pool, which was created by mixing small aliquots of all urine samples. Blank samples were also prepared in the same way as the urine samples, with only the urine being omitted in the process. Each 96-well plate included 4 individually prepared quality control samples and 2 blanks. Further details on the sample preparation can be found in the Supplemental Material.

### Laboratory analysis and preprocessing

Study samples were analyzed using a Q Exactive mass spectrometer with heated electrospray ionization (HESI-II) coupled to a Dionex UltiMate 3000 Binary UHPLC system (ThermoFisher Scientific Inc.). Ten independent analytical batches consisting of 2 individual 96-well plates were analyzed. The mass spectrometer was operated in polarity-switching electrospray ionization mode (positive and negative ionization mode) to expand the coverage of the metabolome.

Preprocessing of the raw data was performed using Compound Discoverer 3.3 software (ThermoFischer Scientific). Metabolomic feature alignment between samples was performed with a maximum retention time window of 0.05 min and a mass tolerance of 5 ppm. Metabolite features were put forward into the feature table only if they were present in ≥2% of the overall samples. Metabolite features present in every blank sample were excluded, unless 5-fold greater in average intensity in samples. Furthermore, metabolite features absent in >95% of the study population were removed, and metabolite features that were missing in ≥30% of consumers (participants who habitually/acutely ate a food) were excluded. For the remaining metabolite features, missing values were imputed with half of the minimum value, that is, intensities, in each analytical batch, assuming missingness due to limits of detection [45]. See the Supplemental Material for more information on the data preprocessing.

### Statistical analyses

All analyses were performed using R 4.2.2 [46], primarily with the following packages: tidyverse (version 1.3.2) [47], nlme (version 3.1-160) [48], and multivariate modeling with minimally biased variable selection in R (MUVR; version 0.0.975) [49]. Before statistical analyses, all metabolite feature variables were log-transformed and *z*-standardized.

MUVR algorithm was used to identify the most predictive metabolite features for the dietary intakes (short-term or habitual) [50]. This algorithm incorporates recursive variable selection within a repeated double cross-validation scheme. MUVR offers approaches for feature selection in the presence of a large number of variables, namely partial least squares (PLSs) and random forest (RF). Both modeling approaches can be performed as either a regression or a classification analysis. Furthermore, MUVR can handle repeated measurements over time; this can be included in the algorithms by providing the subject ID.

For the short-term intake analysis, a classification analysis was performed for which the intake variables were dichotomized (0 for nonconsumers with intake = 0 g and 1 for consumers with intake >0 g). The dichotomization was done because the short-term intake variables showed pronounced right-skewed distributions. For the habitual intake analysis, the intake variables were kept as continuous variables, and regression analysis was applied.

The MUVR analysis had 2 initial steps: *1*) a test run was performed for each food group with PLS and RF with a low number of repetitions (*n*Rep = 6), and *2*) the modeling approach (PLS or RF) with the best performance, that is., highest Q^2^ or lowest number of misclassifications, for the specific food group was selected. Both PLS and RF have been used in biomarker discovery in the past and have strengths and weaknesses [26,50]. However, this initial 2-step approach ensures that the best-performing modeling approach is selected for each specific food group. Furthermore, step 1 provided valuable information on whether PLS or RF could identify predictive metabolite features; if the initial model could not predict intake, that is, negative Q^2^ or zero correctly classified consumers, no further analysis was performed for that food group.

For the main analysis, the parameter configuration was set to *n*Rep = 50 (number of repetitions), varRatio = 0.85 (ratio of variables retained per iteration), and *n*Outer = 8 (number of outer test segments) for all models, in accordance with the recommendations by Shi et al. [50]. We selected metabolite features from the minimal-optimal model, representing the minimal variable set necessary for optimal method performance. Furthermore, after the initial feature selection by RF or PLS, linear mixed models were applied for the final metabolomic feature selection. An unstructured covariance structure was assumed, and the subject ID, unique to each participant, was set as a random effect. In the linear mixed models, each preselected feature (dependent variable) was regressed on the dietary intake variable (independent variable) and adjusted for the covariates age, sex, country, batch, BMI *z*-score, and energy intake. These covariates were selected as a minimal necessary set of individual characteristics; Supplemental Figure 1 shows a directed acyclic graph showing the theoretical framework for the adjustment in the statistical models. To account for multiple hypothesis testing, the Benjamini–Hochberg procedure was applied to control the false discovery rate at 5%, considering results with a *q* value of <0.05 to be statistically significant. In the DONALD cohort, an identical statistical pipeline was applied (Supplemental Material).

### Metabolite annotation

After the final feature selection, the list of metabolite features was sent back to IARC for annotation. The metabolite features were compared with the in-house database of analytical standards with a 10-ppm mass and a retention time tolerance of 0.25 min. in addition, the *m/z* values were searched in the human metabolome database [51] with a 10-ppm mass tolerance. The quality of the chromatographic peaks and spectra was inspected, and the plausibility of database candidates was assessed based on retention time, isotope pattern, adduct formation, and neutral losses. The best matching identities were confirmed by matching the MS/MS spectra and retention time from the metabolite and the corresponding standard. When standards were not available, MS/MS spectra were compared against those in mzCloud or METLIN [52]. The level of identification was determined as proposed by Sumner et al. [53].

## Results

The baseline and follow-up characteristics were stratified by study sample, being presented in Table 1. At baseline, the median age for both study samples was 6.4 y, and almost half of the participants were female. Most of the participants in the short-term intake sample were from Germany, whereas most participants in the habitual intake sample were from Italy at baseline. In total, 11,397 metabolite features were analyzed in negative ionization mode and 16,559 metabolite features in positive ionization mode in the repeated urine samples. After the initial filtration (i.e., removal of features with >95% missing values), 1616 and 1567 metabolite features remained in negative ionization mode and 2055 and 1984 metabolite features in positive ionization mode, for the short-term and habitual intake samples, respectively (Supplemental Figures 2 and 3 show flow diagrams of the filtration process).

**TABLE 1:** Baseline and follow-up characteristics of the study samples from the IDEFICS/I.Family cohort for short-term and habitual dietary intake.

	Habitual dietary intake study sample, *n* = 599			Short-term dietary intake study sample, *n* = 296		
	W1, *n* = 597	W2, *n* = 596	W3, *n* = 595	W1, *n* = 116	W2, *n* = 105	W3, *n* = 223
Median (range)						
Age, y	6.4 (2.1–9.3)	8.4 (4–11.1)	12.3 (8–15.2)	6.4 (2.7–9.3)	8.5 (4.0–11.1)	11.8 (8.2–15.2)
BMI *z*-score	0.42 (−2.79 to 5.07)	0.48 (−3.3 to 4.18)	0.66 (−2.13 to 3.64)	−0.01 (−1.80 to 2.34)	0.13 (−1.75 to 2.64)	0.48 (−2.13 to 3.64)
Energy intake (kcal)^1^	1642 (1136–2409)	1613 (900–2802)	1643 (779–2656)	1462 (213–2426)	1597 (266–3551)	1462 (274–4346)
*n* (%)						
Female	281 (47)	282 (47)	280 (47)	52 (45)	53 (51)	107 (48)
Country^2^						
Italy	288 (48)	287 (48)	288 (48)	3 (3)	7 (7)	46 (21)
Estonia	140 (23)	140 (23)	139 (23)	2 (2)	0	97 (43)
Belgium	12 (2)	12 (2)	12 (2)	10 (9)	3 (3)	6 (3)
Sweden	51 (9)	50 (8)	49 (8)	47 (41)	43 (41)	35 (16)
Germany	62 (10)	63 (11)	63 (11)	53 (46)	45 (43)	23 (10)
Hungary	30 (5)	30 (5)	30 (5)	1 (0.9)	5 (5)	5 (2)
Spain	14 (2)	14 (2)	14 (2)	0	2 (2)	11 (5)

Abbreviations: FFQ, food frequency questionnaire; IDEFICS, Identification and Prevention of Dietary- and Lifestyle-induced Health Effects in Children and Infants; NCI, United States National Cancer Institute; 24-HDR, 24-h dietary recall; 3d-WDR, 3-d weighted dietary record.

1 Usual energy intake calculated from 24-HDR and FFQ data with NCI method; short-term energy intake from 24-HDR.

2 Summed percentage over 100% due to rounding.

### Identification of metabolites in the IDEFICS/I.Family cohort

#### Short-term intake

Table 2 presents an overview of the number of consumers for each food group and time point. After the initial MUVR test runs, the food groups potato crisps and jelly candy were excluded from the main MUVR analysis. Furthermore, for the food groups chocolate and nut spread and ice cream, the negative and positive ionization mode metabolite features were not included in the main MUVR analysis, respectively. For an overview of the test runs and main analysis with the MUVR algorithms, see Supplemental Tables 1 and 2. After MUVR and linear mixed model analyses, while accounting for repeated measures, 16 metabolite features were associated with chocolate candy intake and 8 metabolite features were associated with candy and sweet intake and were issued for annotation. Of the 24 metabolite features, 4 metabolites were annotated, namely theobromine, cyclo(L-prolyl-L-valyl), xanthosine, and octenoylcarnitine. Table 3 provides the overview of the annotated metabolites and Supplemental Tables 3 and 4 an overview of the selected metabolite features that could not be annotated.

**TABLE 2:** Number of short-term consumers of the food groups in the IDEFICS/I.Family cohort by study time points.

Consumers	W1, *n* = 116	W2, *n* = 105	W3, *n* = 223
	*n* (%)		
Chocolate candy	34 (29)	27 (26)	57 (26)
Chocolate and nut spread	18 (16)	8 (8)	20 (9)
Potato crisps	4 (3)	6 (6)	17 (8)
Jelly candy	6 (5)	9 (9)	9 (4)
Candy and sweets	25 (22)	14 (13)	25 (11)
Cakes, puddings, and cookies	59 (51)	54 (51)	86 (39)
Ice cream	13 (11)	10 (10)	21 (9)

**TABLE 3:** Overview of the identified short-term and/or habitual dietary intake-metabolite associations in the IDEFICS/I.Family cohort.

Food group	Intake type^1^	Coefficient^2^	SE	*q*^3^	Metabolite	Ionization mode	Mass	Identification level^4^	Regulation	HMDB ID	Metabolic pathway
Chocolate candy	Short-term	0.01	0.002	0.02	Theobromine	Positive	180.06473	1	Up	0002825	Caffeine metabolism
	Habitual	0.01	0.003	<0.001							
Cakes, puddings, and cookies	Habitual	0.01	0.002	0.01							
Chocolate candy	Short-term	0.01	0.002	0.01	Xanthosine	Positive	284.07565	1	Up	0000299	Purine metabolism
	Habitual	0.01	0.003	0.003		Negative	284.07584				
Chocolate candy	Short-term	0.01	0.002	0.03	Cyclo(L-prolyl-L-valyl)	Positive	196.12143	1	Up	0240493	NA
	Habitual	0.02	0.003	<0.001							
Candy and sweets	Short-term	-0.01	0.003	0.04	Octenoylcarnitine	Positive	285.19403	2	Down	NA	NA

1 Short-term: dietary intake 1 or 2 d before urine collection; habitual: dietary intake calculated with the United States National Cancer Institute method.

2 Coefficients are on the log and *z* scale and adjusted for age, sex, country, batch, BMI *z*-score, and energy intake.

3 The *q* value is a *p* value that has been adjusted for the false discovery rate.

4 Identification level, see Sumner et al. [53].

The first sensitivity analysis showed an association between chocolate candy intake and theobromine and cyclo(L-prolyl-L-valyl), but not with xanthosine, and between candy and sweets intake and octenoylcarnitine. Additionally, some new metabolite features were identified for chocolate candy, jelly candy, chocolate and nut spread, candy and sweets, and cakes, puddings and cookies (Supplemental Tables 5 and 6). The second sensitivity analysis showed no attenuation of the associations between chocolate candy intake and theobromine, cyclo(L-prolyl-L-valyl), and xanthosine (results not shown).

#### Habitual intake

Table 4 depicts an overview of the median intake in grams per day for each food group and time point. After the initial MUVR test runs, the food group savory and fatty snacks was not selected for the main MUVR analysis. Results of the test runs and main analysis with the MUVR algorithms are provided in the Supplemental Tables 7 and 8. We accounted for repeated measures during MUVR and linear mixed model runs. The analyses showed that 45 metabolite features were associated with chocolate candy intake; 5 metabolite features were associated with candy and sweet intake; 1 metabolite feature was associated with crisp intake; 9 metabolite features were associated with the intake of cakes, puddings, and cookies; and 2 metabolite features were associated with ice cream intake. Of these metabolite features, 3 metabolites could be annotated. Same as for short-term intake, habitual intake of chocolate candy was associated with theobromine, cyclo(L-prolyl-L-valyl), and xanthosine. Additionally, intake of cakes, puddings, and cookies was associated with theobromine. Table 3 presents the overview of identified and annotated metabolites and Supplemental Tables 3 and 4 an overview of the selected metabolite features that could not be annotated.

**TABLE 4:** Median habitual dietary intake of the food groups in the IDEFICS/I.Family cohort by study time points.

Dietary intake (g/d)	W1, *n* = 597	W2, *n* = 596	W3, *n* = 595
	Median (range)		
Chocolate candy	7.8 (1.2–55.6)	6.8 (2.1–52.2)	11.4 (2.7–71.2)
Savory and fatty snacks	62.1 (30.3–119.4)	70.2 (2.5–153.8)	63.2 (21.2–153.7)
Candy and sweets	0.6 (0.2–27.1)	3.1 (0.5–21.4)	1.4 (0.1–39.8)
Crisps	1.3 (0.4–138.9)	1.2 (0.3–119.7)	6.2 (2.4–42.4)
Cakes, puddings, and cookies	65.3 (34.8–99.2)	60.8 (19.4–121.4)	55.0 (24.5–140.2)
Ice cream	4.1 (1.4–152.5)	16.0 (0.9–204.1)	11.9 (4.4–70.4)

### Replication of metabolites in the DONALD cohort

During the initial MUVR analysis, only chocolate candy intake was moved forward to the final metabolite feature selection. Supplemental Table 9 presents an overview of the individual characteristics and the median intake for chocolate candy in the DONALD replication cohort.

In the independent DONALD cohort, chocolate candy intake was associated with 45 metabolite features. Of these metabolite features, 4 were annotated, namely theobromine, cyclo(L-prolyl-L-valyl), xanthosine, and 3-hydroxyphenylacetate (Table 5).

**TABLE 5:** Overview of the annotated metabolites measured in negative and positive ionization mode associated with chocolate intake in the DONALD cohort—results from the replication analysis.

Food group	Coefficient^1^	SE	*q*^2^	Metabolite	Ionization mode	Mass	Identification level^3^	Regulation
Chocolate candy	0.02	0.003	<0.001	Theobromine	Positive	180.06467	1	Up
Chocolate candy	0.02	0.003	<0.001	Cyclo(L-prolyl-L-valyl)	Positive	196.12136	1	Up
Chocolate candy	0.01	0.003	<0.001	Xanthosine^4^	Positive	366.14252	1	Up
Chocolate candy	0.01	0.003	0.001	Xanthosine^4^^,^^5^	Positive	284.07553	1	Up
Chocolate candy	0.01	0.003	0.003	Xanthosine^4^	Positive	244.14232	1	Up
Chocolate candy	0.01	0.003	0.001	3-hydroxyphenylacetate	Negative	152.04745	2	Up
Chocolate candy	0.01	0.003	0.01	Xanthosine^4^	Negative	366.14261	1	Up
Chocolate candy	0.01	0.003	0.03	Xanthosine^4^^,^^5^	Negative	284.07574	1	Up

1 Coefficients are on the log scale and adjusted for age, sex, BMI, and energy intake.

2 The *q* value is a *p* value that has been adjusted for the false discovery rate.

3 Identification level, see Sumner et al. [53].

4 Metabolite features belong to the same metabolite.

5 Main metabolite.

## Discussion

Using data from a large European cohort of children and adolescents, we were able to identify potential biomarkers of short-term and habitual intake of sweet and fatty snacks. Importantly, 3 putative biomarkers of chocolate intake, namely theobromine, xanthosine, and cyclo(L-prolyl-L-valyl), were externally replicated in the German DONALD cohort.

Most studies in children have focused on identifying biomarkers of fruit and vegetable intake. Furthermore, many studies have used cross-sectional data or data from dietary interventions for biomarker identification [25]. Only 2 studies have used cohort data to identify BFIs in children [24,54]. Our study used longitudinal cohort data with the repeatedly measured urine metabolome to identify biomarkers of fatty and sweet snack intake in children. This approach highlights the novelty of this study and can contribute to the further use of cohort data in the field of biomarker identification.

Before discussing the results, it is important to highlight the main challenges of our methodologic approach. The analyses were based on large cohort studies but still depended on self-reported dietary intake data. In this study, the 24-HDR and FFQ were used to assess the dietary intake of the participants of the main cohort, methods often criticized for their questionable validity in accurately reflecting children’s diets [4]. The 24-HDR, used as short-term intake measurement, likely introduces a bias toward the null, because sweet and fatty snack intakes might be underreported [4,55]. Nevertheless, efforts were made to validate the 24-HDR and FFQ, enhancing accurate dietary assessment [33,35,38]. Furthermore, the combination of 24-HDR and FFQ data in this study reduces biases and increases the accuracy of individual habitual dietary intake estimates [56,57]. Our approach, however, would not be suitable to capture biomarkers of rarely consumed foods, which would require feeding studies. However, this study focused on regularly consumed snacks. Only biomarkers with longer half-lives and foods frequently consumed would be useful for cohort studies that typically rely on single urine sample collections. We studied acute intake biomarkers as a proof of principle by linking 24-HDR with urine samples from the previous day. The successful replication of 3 biomarker candidates can also be seen as a proof of concept for our methodologic approach.

In this study, besides the 4 annotated metabolites, 62 unique metabolite features were selected but could not be annotated. Unfortunately, it is possible neither to provide any further information on the identity of these metabolite features nor to assess biological plausibility. This is common in untargeted metabolomics analysis due to the vast number of features detected by mass spectrometry [58]. Additionally, ∼60 more unknowns were selected in the sensitivity analysis. The mass of these unknowns is provided in the Supplemental Material for future studies to use for reference.

The candy and sweets–related metabolite in the discovery cohort, octenoylcarnitine, belongs to the class of acylcarnitines and is a medium-chain acylcarnitine. Increased concentrations of octenoylcarnitine are associated with obesity and fatty acid metabolism disorders [59]. Only 2 studies have reported associations between diet and octenoylcarnitine [60,61]. In this study, we found a negative association between candy and sweet intake and octenoylcarnitine. According to the validation criteria by Dragsted et al. [62], a metabolite should increase in response to dietary intake, that is, there should be a positive association with dietary intake. Therefore, octenoylcarnitine is unlikely a relevant BFI of candy and sweet intake in our cohort.

Theobromine is an organic compound and belongs to the class of xanthines [63]. In humans, this metabolite is a product of caffeine breakdown by CYP1A2 in the liver [64]. Measured in blood, theobromine has a half-life of 6–8 h. Theobromine is found in the highest concentrations in cocoa products and in smaller concentrations in coffee and tea [63,65]. Accordingly, many studies have identified theobromine as a potential BFI of cocoa products [17]. However, Michielsen et al. [17] dismissed theobromine as a potential BFI of cocoa products, arguing that it is not specific to the consumption of cocoa products. Indeed, in our study, theobromine was also associated with intake of cakes, puddings, and cookies, but this may reflect the cocoa ingredients in these snacks.

Xanthosine is a purine nucleoside and an intermediate in the purine metabolism [66]. It is produced during the breakdown of theobromine, which is derived from caffeine [67]. Xanthosine is expected to be present in cocoa beans, and other foods but has not been quantified in these until now [66]. Several studies have associated methylxanthines, such as 7-methylxanthosine, which is the precursor of xanthosine, with cocoa (products) consumption [17]. However, our literature search found no evidence linking chocolate intake and xanthosine in previous literature.

Cyclo(L-prolyl-L-valyl) belongs to the class of α-amino acids and derivatives [68]. Few studies have been published about this metabolite. Nonetheless, it is linked to cocoa (products) and coffee intake [69]. One study found a correlation between cyclo(L-prolyl-L-valyl) and chocolate intake [70], whereas another study found a correlation between cyclo(L-prolyl-L-valyl) and habitual coffee intake [71]. Beyond these findings, we could not identify any study that reported an association between chocolate or cocoa (products) and cyclo(L-prolyl-L-valyl).

Theobromine, xanthosine, and cyclo(L-prolyl-L-valyl) may be potential candidate biomarkers of coffee and/or black tea intake, making them unsuitable for identifying chocolate intake. However, this issue is less relevant for younger children, who are typically nonconsumers of coffee and black tea, although it may be relevant for adolescents. A sensitivity analysis was performed by adding short-term coffee and tea intake as a covariate into the linear mixed model, which did not alter the results of the short-term chocolate intake analysis. Only very few children reported consuming coffee or tea (W1, *n* = 16; W2, *n* = 16; and W3, *n* = 55).

Notably, Europe is among the largest chocolate consumers globally, followed closely by the United States [72]. According to the European Food Safety Agency, chocolate (beverages) is the main source of caffeine intake for children aged 3–10 y [73]. Therefore, these BFIs could be valuable in assessing chocolate intake in children. From a public health perspective, the detection of metabolites from caffeine pathways in children consuming chocolate may be controversial. Future studies should evaluate the half-life and pharmacologic properties of xanthines like theobromine for children.

Indeed, the elimination half-life of the metabolites might be an important aspect. In the first sensitivity analysis, we restricted the short-term sample to participants with a 1-d interval between urine collection and dietary intake. We could identify theobromine, cyclo(L-prolyl-L-valyl), and octenoylcarnitine, as well as additional metabolites for 6 food groups not found in the main analysis. The closer timing between dietary intake and urine collection enabled us to detect associations between short-term dietary intake and metabolites that might have been cleared in those with a longer interval between intake and collection.

All 3 chocolate metabolites were replicated in the DONALD cohort, adding to the evidence that theobromine, xanthosine, and cyclo(L-prolyl-L-valyl) are potential biomarkers of chocolate intake in children and adolescents. In addition, 3-hydroxyphenylacetate, a metabolite not previously identified as a chocolate intake biomarker, was found in the replication cohort. The differences between the main and replication cohort, such as dietary assessment methods (24-HDR compared with 3d-WDR), urine collection modes (morning urine compared with 24h-urine), and country of residence (European countries compared with Germany), could explain the different results. The 24-h urine collection may present higher validity and could capture a wider range of metabolites, whereas the timing of morning urine samples might miss some metabolites due to their elimination half-life [74]. Although 1 study suggested only small differences between the applied dietary assessment methods, the impact on the study outcome is still uncertain [75]. Furthermore, the country of residence may be an important factor and explains a part of the variance in the urine metabolome [24,76].

Lastly, we could not identify potential BFIs for all snack food groups we investigated. This may be due to the nonspecific nature of some of the food groups, for example, combining cakes, puddings, and cookies into 1 group; and the low number of consumers for certain items, like jelly candy. For an in-depth study of biomarkers of other snack foods, a more specific dietary assessment of the nature, ingredients, and brands of these convenience snack foods, along with higher sample sizes, would be recommended.

That said, the limitations of this study must be highlighted: The main limitation is the reliance on self-reported dietary intake, as discussed in the beginning. Additionally, the half-life of elimination may have affected the short-term analysis, where metabolite features were found for only 2 of 7 food groups. Although we sought to identify the best statistical pipeline for the available data, the use of machine learning (MUVR algorithms), and standard statistical techniques (linear mixed models) is not common practice in biomarker discovery [26,77]. Furthermore, the differential analysis mode for short-term intake (dichotomization and classification analysis) and habitual intake (continuous and regression analyses) could have influenced the number of metabolites related to short-term and habitual intake, although the number of features in both analyses was comparable.

This study has several strengths. We made use of 2 independent longitudinal cohorts of European children, which allowed us to evaluate the consistency of the results. The 3 chocolate metabolites were identified by a 2-step statistical analysis strategy, which was also applied in the DONALD cohort. The application of repeated double crossvalidation with unbiased variable selection algorithms provided a stable selection of metabolite features in the face of a large number of initial metabolite features [50]. Additionally, we applied linear mixed models to account for important individual characteristics. In both cohorts, the whole (food) metabolome was measured by untargeted metabolomics, covering a large number of available metabolites in the urine samples. Repeatedly measured food metabolome data were available in both cohorts. The laboratory and statistical analysis pipelines were streamlined for both cohorts, making the analysis approaches as identical as possible. Combining the FFQ and 24-HDR data with the NCI method potentially increased the precision of the predicted habitual dietary intakes over merely averaging dietary intakes from the repeated 24-HDR [40].

In conclusion, in this study, we analyzed untargeted metabolomics data measured in repeatedly collected urine samples. We were able to identify and replicate theobromine, xanthosine, and cyclo(L-prolyl-L-valyl) in 2 independent cohorts of children and adolescents. This approach is novel and demonstrates the potential use of cohort data for the identification of biomarkers. Nonetheless, further research is needed to assess the validity and specificity of these potential chocolate BFIs in children, especially in light of coffee and tea intake. Controlled feeding studies with targeted metabolomics measurements would be needed for further validation.

## Author contributions

The authors’ responsibilities were as follows – AF, UN, IH, PK-R, JR: designed research; SDH, MH, IH, LL, DM, LAM, PR, TV, UN: conducted research; JR, MM, DA, PK-R: performed laboratory analysis, metabolomics data preprocessing, and metabolite annotation; JG: performed the statistical analysis in the discovery cohort; LY: made significant contributions toward the statistical analysis in the discovery cohort; RF: provided guidance on the statistical analysis in the discovery cohort; SM: performed statistical analysis in the replication cohort; KO: revised and supervised statistical analysis in the replication cohort; JG: wrote paper; AK: supervised and made significant contributions to the writing process of the paper; JG, AF: had primary responsibility for final content; and all authors: have read and approved the final manuscript.

## Funding

This study was funded by the German Research Foundation (DFG project number 406710821) and the Agence Nationale de la Recherche (ANR project number ANR-18-CE92-0060). This work was done as part of the IDEFICS (http://www.idefics.eu) and I.Family studies (http://www.ifamilystudy.eu/) and the DONALD study. We gratefully acknowledge the financial support of the European Commission within the Sixth RTD Framework Programme Contract No. 016181 (FOOD), and the Seventh RTD Framework Programme Contract No. 266044. The DONALD study is financially supported by the Ministry of Science and Research of North Rhine-Westphalia, Germany.

## Data availability

Data described in the manuscript, code book, and analytic code will be made available upon request pending application and approval.

## Disclaimer

Where authors are identified as personnel of the International Agency for Research on Cancer/World Health Organization, the authors alone are responsible for the views expressed in this article and they do not necessarily represent the decisions, policy or views of the International Agency for Research on Cancer /World Health Organization.

## Conflict of interest

AF reports financial support was provided by German Research Foundation. PK-R reports financial support was provided by French National Research Agency. All other authors report no conflicts of interest.
