# Supplementary material for: Identification and Replication of Urine Metabolites Associated With Short-Term and Habitual Intake of Sweet and Fatty Snacks in European Children and Adolescents
Source: J Nutr. 2024 Sep 25;154(11):3274–85. doi: 10.1016/j.tjnut.2024.09.026 (PMC11600116; doi:10.1016/j.tjnut.2024.09.026)
Supplement: multimedia component 2 [file mmc2.pdf]

Supplementary Material

Identification and replication of urine metabolites associated with short-term and habitual intake of sweet and fatty snacks in European children and adolescents

Jantje Goerdten[1], Samuel Muli[2], Jodi Rattner[3], Mira Merdas[3], David Achaintre[3], Li Yuan[1], Stefaan De Henauw[4], Ronja Foraita[1], Monica Hunsberger[5], Inge Huybrechts[3], Lauren Lissner[5], Dénes Molnár[6], Luis A. Moreno[7,8], Paola Russo[9], Toomas Veidebaum[10], Krasimira Aleksandrova[1,11], Ute Nöthlings[2], Kolade Oluwagbemigun[2], Pekka Keski-Rahkonen[3]* & Anna Floegel[1,12]*

[1]Leibniz Institute for Prevention Research and Epidemiology (BIPS), Bremen, Germany

[2]Unit of Nutritional Epidemiology, Department of Nutrition and Food Sciences, University of Bonn, Bonn, Germany

[3]International Agency for Research on Cancer (IARC), Lyon, France

[4]Department of Public Health and Primary Care, Ghent University, Ghent, Belgium

[5]School of Public Health and Community Medicine, Institute of Medicine, Sahlgrenska Academy, University of Gothenburg, Gothenburg, Sweden

[6]Department of Pediatrics, Medical School, University of Pécs, Pécs, Hungary

[7]GENUD (Growth, Exercise, NUtrition and Development) Research Group, Faculty of Health Sciences, University of Zaragoza, Instituto Agroalimentario de Aragón (IA2) and Instituto de Investigación Sanitaria Aragón (IIS Aragón), Zaragoza, Spain

[8]Consorcio CIBER, M.P. Fisiopatología de la Obesidad y Nutrición (CIBERObn), Instituto de Salud Carlos III (ISCIII), Madrid, Spain

[9]Institute of Food Sciences, CNR, Avellino-Italy

[10]National Institute for Health Development, Tallinn, Estonia

[11]Faculty of Human and Health Sciences, University of Bremen, Bremen, Germany

[12]Section of Dietetics, Faculty of Agriculture and Food Sciences, Hochschule Neubrandenburg - University of Applied Sciences, Neubrandenburg, Germany

*equally contributed to this work.

Laboratory analysis for IDEFICS/I.Family

Sample Preparation

Samples (n=1800) were prepared by diluting 30 uL of urine with ultra-pure water based on normalization to lowest specific gravity in all samples (IDEFICS: 1.008). Then 30 uL of the diluted urine samples were mixed with 270 uL of cold acetonitrile in Agilent Captiva 96 Deep Well plates (Agilent Technologies France; ref: A696001000B). The precipitate was filtered and 100 uL was transferred to Thermo Well 96 plates (Thermo Electron SAS; ref: 6820-4100) . The plate was immediately sealed with a rapid EPS adhesive plate sheet (Teknolab Sorbent;ref: BC-REPS001) and analysed. Quality control (QC) samples were prepared from a sample pool that was made by mixing small aliquots of all samples and extracted along with the study samples. Blank samples were also prepared along the urine samples in an identical manner, only leaving out urine in the process. Each well plate included four individually prepared QCs and two blanks.

Sample Analysis

Samples were analysed as 10 independent analytical batches consisting of 2 individual 96-well plates. The repeated samples were analysed next to each other in random order, and sample pairs were randomized across the batch. A UHPLC-QE-MS system was used that consisted of a Dionex UltiMate 3000 Binary LC system, and a Q-Exactive mass spectrometer with heated electrospray ionization (HESI-II) (Thermo Scientific). Samples were kept at 5°C and 2 µL was injected. An ACQUITY UHPLC HSS T3 column (2.1 × 100mm, 1.8 µm; Waters) was used at 45 °C and the mobile phase consisted of ultrapure water and LC-MS grade methanol, both with 0.05 % (v/v) of formic acid. The gradient profile was as follows: 0–6 min: 5% to 100% methanol, 6–10.5 min: 100% methanol, 10.5–13 min: 5% methanol. The flow rate was 0.4 ml/min.

The mass spectrometer was operated in a positive/negative switching polarity using the following conditions: spray voltage 4.0 kV, sheath gas flow rate 50 (Arbitrary unit; A.u), auxillary gas flow rate 13 (A.u), sweep gas flow rate 3 (A.u), Aux gas heater temperature 425°C, capillary temperature 260°C and a S-Lens RF level 60%. For the analysis a full MS scan mode over a mass range of 66.7 to 1000 Da, at a resolution 35000 with an associated scan rate at 2.1Hz. AGC target 1e6 and a maximum injection time 50 ms was applied. MS/MS analyses were performed on QC samples with an isolation width of 2.0 Da, in positive and negative modes at 3 normalized collision energies 30, 60 and 90. Data was acquired in centroid format.

Data Processing

Pre-processing was performed using Compound Discoverer 3.3 software (Thermo Fisher Scientific). A minimum peak intensity threshold and mass tolerance of 500 000 and 5 ppm respectively were used to find [M+H]+ and [M-H]- ions in positive and negative mode data, respectively. Feature alignment between samples was performed with maximum retention time window of 0.05 min and mass tolerance of 5ppm. Features were put forward into the feature table only if they were present in at least 2% of the overall samples. Features present in every

blank sample were excluded, unless 5-fold greater in average intensity in samples. The final feature table was exported as a .xlsx file. Peak areas were used as a measurement of intensity.

Quality Control

Quality control was performed using data from the QC samples. The assessment was based on the following attributes:

- Response stability: in chronological order, area median response of features found in all QC samples.
- Response variability: distribution of MS features according to their Relative Standard Deviations (RSD%) of features found in all QC samples.
- Response variability: RSD% of 10 known compounds in all QC samples.

Laboratory analysis for DONALD

Sample Preparation

Samples (n=600) were prepared by diluting 30 uL of urine with ultra-pure water based on normalization to lowest specific gravity in all samples (DONALD: 1.079). Then 30 uL of the diluted urine samples were mixed with 270 uL of cold acetonitrile in Agilent Captiva 96 Deep Well plates (Agilent Technologies France; ref: A696001000B). The precipitate was filtered and 100 uL was transferred to Thermo Well 96 plates (Thermo Electron SAS; ref: 6820-4100). The plate was immediately sealed with a rapid EPS adhesive plate sheet (Teknolab Sorbent; ref: BC-REPS001) and analysed. Quality control (QC) samples were prepared from a sample pool that was made by mixing small aliquots of all samples and extracted along with the study samples. Blank samples were also prepared along the urine samples in an identical manner, only leaving out urine in the process. Each well plate included four individually prepared QCs and two blanks.

Sample Analysis

Samples were analysed as 4 independent analytical batches consisting of 2 individual 96-well plates. The repeated samples points were analysed next to each other in random order, and sample pairs were randomized across the batch. A UHPLC-QE-MS system was used that consisted of a Dionex UltiMate 3000 Binary LC system, and a Q-Exactive mass spectrometer with heated electrospray ionization (HESI-II) (Thermo Scientific). Samples were kept at 5°C and 2 µL was injected. An ACQUITY UHPLC HSS T3 column (2.1 × 100mm, 1.8 µm; Waters) was used at 45 °C and the mobile phase consisted of ultrapure water and LC-MS grade methanol, both with 0.05 % (v/v) of formic acid. The gradient profile was as follows: 0–6 min: 5% to 100% methanol, 6–10.5 min: 100% methanol, 10.5–13 min: 5% methanol. The flow rate was 0.4 ml/min.

The mass spectrometer was operated in a positive/negative switching polarity using the following conditions: spray voltage 4.0 kV, sheath gas flow rate 50 (Arbitrary unit; A.u), auxillary gas flow rate 13 (A.u), sweep gas flow rate 3 (A.u), Aux gas heater temperature 425°C, capillary temperature 260°C and a S-Lens RF level 60%. For the analysis a full MS scan mode over a mass range of 66.7 to 1000 Da, at a resolution 35000 with an associated scan rate at 2.1Hz. AGC target 1e6 and a maximum injection time 50 ms was applied. MS/MS analyses were performed on QC samples with an isolation width of 2.0 Da, in positive and negative modes at 3 normalized collision energies 30, 60 and 90. Data was acquired in centroid format.

Data Processing

Pre-processing was performed using Compound Discoverer 3.3 software (Thermo Fisher Scientific). A minimum peak intensity threshold and mass tolerance of 500 000 and 5 ppm respectively were used to find [M+H]+ and [M-H]- ions in positive and negative mode data, respectively. Feature alignment between samples was performed with maximum retention time window of 0.05 min and mass tolerance of 5ppm. Features were put forward into the feature table only if they were present in at least 2% of the overall samples. Features present in every blank sample were excluded, unless 5-fold greater in average intensity in samples. The final feature table was exported as a .xlsx file. Peak areas were used as a measurement of intensity.

Quality Control

Quality control was performed using data from the QC samples. The assessment was based on the following attributes:

- Response stability: in chronological order, area median response of features found in all QC samples.
- Response variability: distribution of MS features according to their Relative Standard Deviations (RSD%) of features found in all QC samples.
- Response variability: RSD% of 10 known compounds in all QC samples.

Statistical Analysis in DONALD

In the replication sample the PLS algorithm incorporated in MUVR was applied. The PLS model (run as a regression analysis) was tuned according to Shi et al. [1] recommendations. The final model had varRatio = 0.90, nOuter = 8, and was repeated 50 times for stability of selections and ranking (nRep = 50). Only the features from the minimal model were further analysed in the linear mixed model. In the linear mixed model, the dietary intake variable was set as the independent variable and the selected features as dependent variable. Covariates were entered into the linear mixed models, including age, sex, BMI, and energy intake. The Benjamini-Hochberg procedure to control the False Discovery Rate (FDR) at 5% was applied, and accepting results with $q < 0.05$ as statistically significant.

1. Shi, L., et al., *Variable selection and validation in multivariate modelling.* Bioinformatics, 2019. **35**(6): p. 972-980.

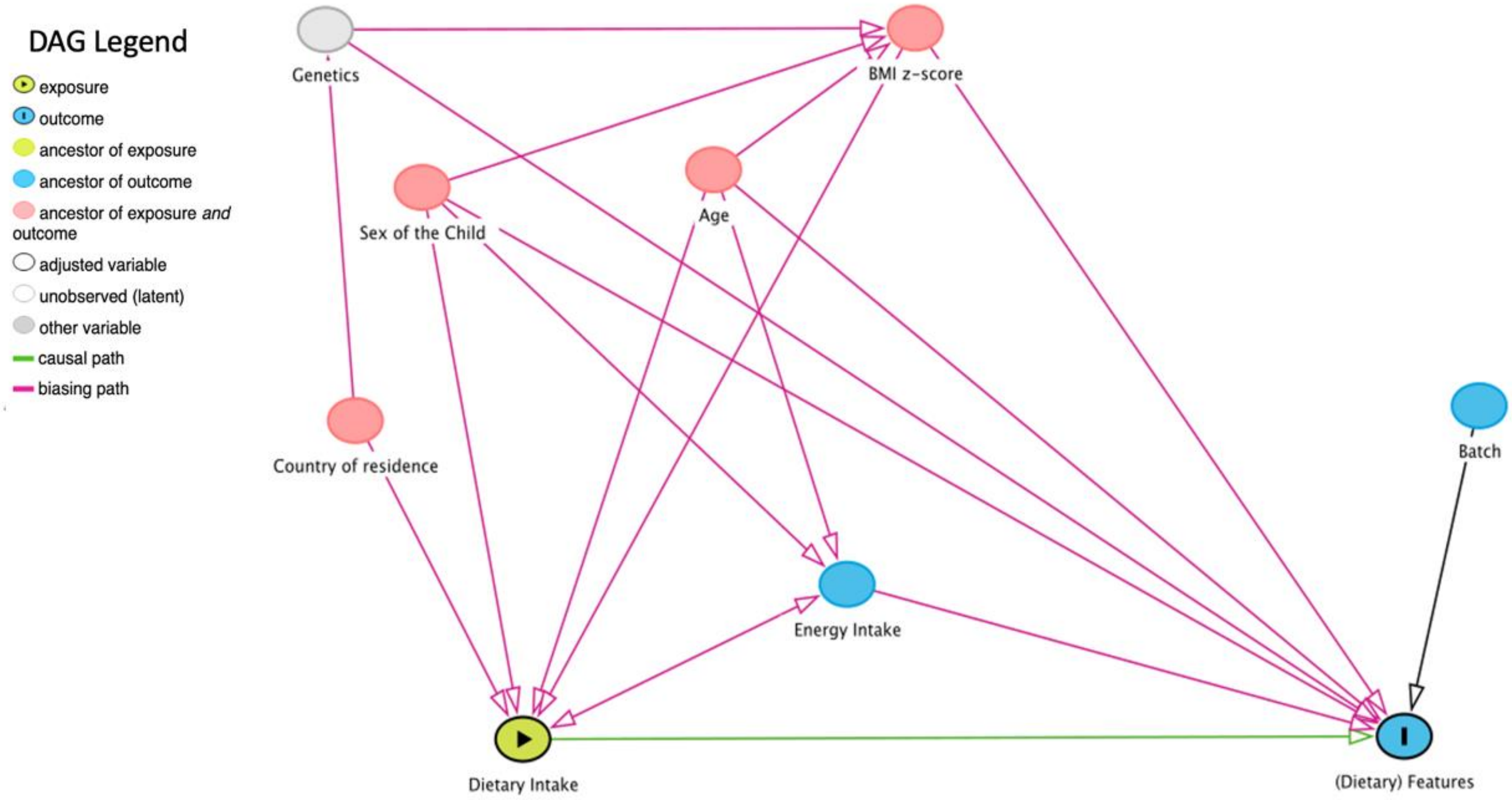

Supplementary Figure 1: Directed Acyclic Graph: a theoretical framework for the adjustments in the linear mixed models for the short-term and habitual intake samples from the IDEFICS/I.Family cohort

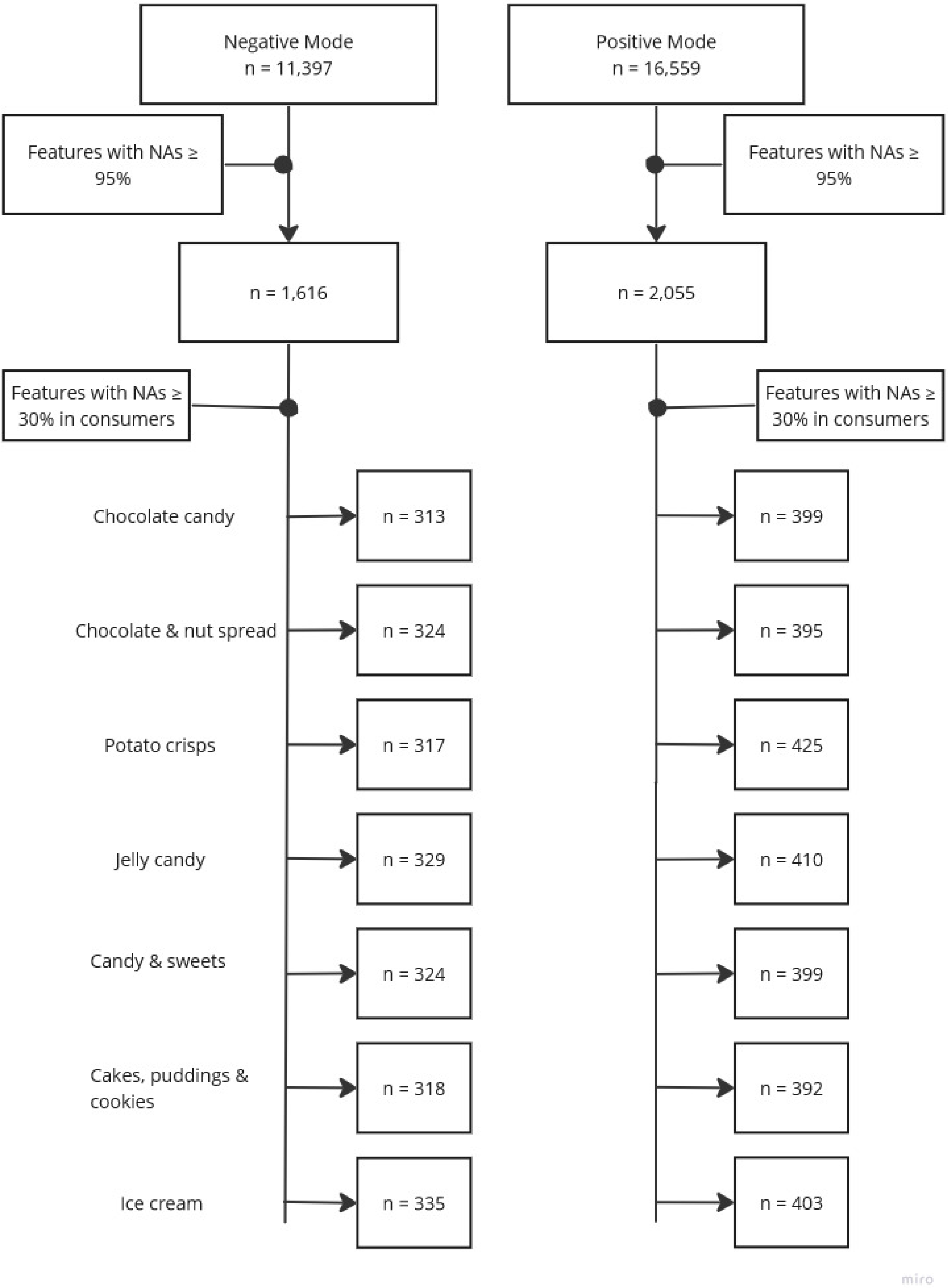

Supplementary Figure 2: Flow diagram of the feature exclusion in the short-term intake sample from the IDEFICS/I.Family cohort

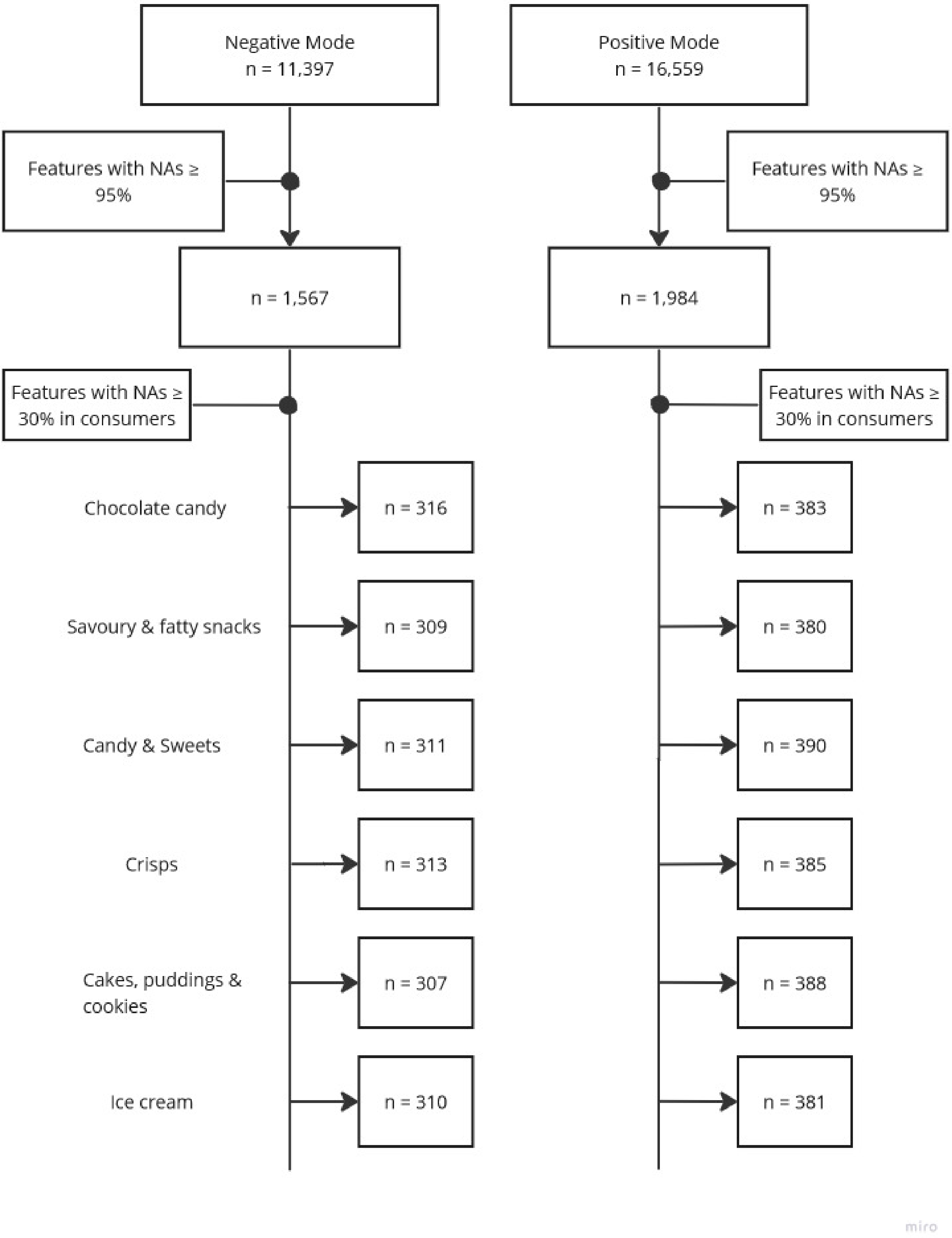

Supplementary Figure 3: Flow diagram of the feature exclusion in the habitual intake sample from the IDEFICS/I.Family cohort

Supplementary Table 1 Overview of the repeated double cross-validation with unbiased variable selection (MUVR) test runs on the short-term dietary intake food groups from IDEFICS/I.Family.

Food Group	Ionization mode	Method	Prediction Fitness Classification[1]
Chocolate candy	Negative	RF	11%
		PLS	23%
	Positive	RF	17%
		PLS	17%
Chocolate & nut spread	Negative	RF	0%
		PLS	0%
	Positive	RF	0%
		PLS	2%
Potato crisps	Negative	RF	0%
		PLS	0%
	Positive	RF	0%
		PLS	0%
Jelly candy	Negative	RF	0%
		PLS	0%
	Positive	RF	0%
		PLS	0%
Candy & sweets	Negative	RF	9%
		PLS	9%
	Positive	RF	1%
		PLS	1%
Cakes, puddings & cookies	Negative	RF	50%
		PLS	46%
	Positive	RF	52%
		PLS	52%
Ice cream	Negative	RF	2%
		PLS	0%
	Positive	RF	0%
		PLS	0%

[1]The prediction fitness for the classification analysis provided by the MUVR package was replaced by a new function to calculate the percentage of correctly classified consumers in the sample.

Supplementary Table 2 Results of main classification analysis with repeated double cross-validation with unbiased variable selection (MUVR) algorithms on the short-term dietary intake sample from the IDEFICS/I.Family study.

Food Group	Ionization mode	Algorithm	Original variables	Minimal optimal[2]	Prediction fitness Classification[3]
Chocolate candy	Negative	PLS	313	43	22%
	Positive	PLS	399	45	21%
Chocolate & nut spread	Negative	-[1]	324	-[1]	-[1]
	Positive	PLS	395	124	0%
Potato crisps	Negative	-[1]	317	-[1]	-[1]
	Positive	-[1]	425	-[1]	-[1]
Jelly candy	Negative	-[1]	329	-[1]	-[1]
	Positive	-[1]	410	-[1]	-[1]
Candy & sweets	Negative	RF	324	12	9%
	Positive	PLS	399	90	8%
Cakes, puddings & cookies	Negative	RF	318	21	47%
	Positive	RF	392	15	50%
Ice cream	Negative	RF	335	8	0%
	Positive	-[1]	403	-[1]	-[1]

[1]After test-run removed from main analysis.

[2]Number of variables selected as minimal-optimal using MUVR 'min' model.

[3]The prediction fitness for the classification analysis provided by the MUVR package was replaced by a new function to calculate the percentage of correctly classified consumers in the sample.

Supplementary Table 3 Overview of the features measured in positive ionization mode without annotation associated with short-term and/or habitual dietary intake in IDEFICS/I.Family.

<table>
<tr><th>Food Group</th><th>Intake Type</th><th>Coefficient[1]</th><th>SE</th><th>p-value[2]</th><th>Mass</th><th>Retention time</th><th>m/z</th><th>Regulation</th></tr>
<tr><td>Chocolate Candy</td><td rowspan="2">Habitual</td><td>-0.01</td><td>0.002</td><td>0.04</td><td rowspan="2">100.01603</td><td rowspan="2">1.345</td><td rowspan="2">101.02331</td><td>Down</td></tr>
<tr><td>Cakes, puddings & cookies</td><td>0.003</td><td>0.001</td><td>0.02</td><td>Up</td></tr>
<tr><td>Chocolate Candy</td><td>Short-term</td><td>0.01</td><td>0.002</td><td>0.01</td><td>182.04404</td><td>1.795</td><td>183.05132</td><td>Up</td></tr>
<tr><td rowspan="2">Chocolate Candy</td><td>Short-term</td><td>0.01</td><td>0.002</td><td>0.02</td><td rowspan="3">166.04917</td><td rowspan="3">1.896</td><td rowspan="3">167.05645</td><td rowspan="2">Up</td></tr>
<tr><td>Habitual</td><td>0.01</td><td>0.003</td><td><0.001</td></tr>
<tr><td>Cakes, puddings & cookies</td><td>Habitual</td><td>0.01</td><td>0.002</td><td>0.003</td><td>Up</td></tr>
<tr><td>Chocolate Candy</td><td>Short-term</td><td>0.01</td><td>0.002</td><td>0.04</td><td>150.05437</td><td>2.037</td><td>151.06165</td><td>Up</td></tr>
<tr><td>Chocolate Candy</td><td>Habitual</td><td>0.01</td><td>0.003</td><td><0.001</td><td>119.03725</td><td>2.458</td><td>120.04453</td><td>Up</td></tr>
<tr><td>Chocolate Candy</td><td>Short-term</td><td>0.01</td><td>0.002</td><td>0.01</td><td>170.10564</td><td>2.729</td><td>171.11292</td><td>Up</td></tr>
<tr><td>Chocolate Candy</td><td>Short-term</td><td>-0.01</td><td>0.002</td><td>0.02</td><td>286.0927</td><td>3.098</td><td>287.09998</td><td>Down</td></tr>
<tr><td>Chocolate Candy</td><td rowspan="2">Habitual</td><td>0.02</td><td>0.003</td><td><0.001</td><td rowspan="2">274.09872</td><td rowspan="2">3.403</td><td rowspan="2">275.106</td><td>Up</td></tr>
<tr><td>Candy & sweets</td><td>-0.02</td><td>0.005</td><td>0.01</td><td>Down</td></tr>
<tr><td>Chocolate Candy</td><td>Short-term</td><td>0.01</td><td>0.002</td><td>0.02</td><td>191.05823</td><td>3.487</td><td>192.06551</td><td>Up</td></tr>
</table>

	Habitual	0.01	0.003	<0.001				
Chocolate Candy	Short-term	0.01	0.002	0.02	401.27762	5.073	402.2849	Up
Candy & sweets	Habitual	0.02	0.005	0.002	260.09095	1.467	261.09823	Up
Candy & sweets	Short-term	-0.01	0.004	0.03	243.14718	2.54	244.15446	Down
Candy & sweets	Habitual	-0.01	0.005	0.04	351.09536	3.379	352.10264	Down
Candy & sweets	Short-term	-0.01	0.003	0.03	283.17839	3.549	284.18567	Down
Candy & sweets	Short-term	-0.02	0.004	0.01	313.22533	4.951	314.23261	Down
Crisps	Habitual	0.01	0.002	0.03	332.06773	2.664	333.07501	Up
Cakes, puddings & cookies	Habitual	0.01	0.002	0.01	244.1424	1.908	245.14968	Up
Cakes, puddings & cookies	Habitual	0.01	0.001	0.01	261.08492	2.016	262.0922	Up
Cakes, puddings & cookies	Habitual	0.004	0.002	0.03	210.10068	2.111	211.10796	Up
Cakes, puddings & cookies	Habitual	0.004	0.001	0.01	248.05869	5.433	249.06597	Up
Ice Cream	Habitual	0.002	0.001	0.04	173.1165	1.526	174.12378	Up
Ice Cream	Habitual	0.002	0.001	0.003	173.1165	0.95	174.12378	Up

[1]Coefficient are on the log and z scale and adjusted for age, sex, country, batch, BMI z-score and energy intake.
[2]p-value adjusted for false discovery rate (FDR).

Supplementary Table 4 Overview of the features measured in negative ionization mode without annotation associated with short-term and/or habitual dietary intake in IDEFICS/I.Family.

Food Group	Intake Type	Coefficient[1]	SE	p-value[2]	Mass	Retention time	m/z	Regulation
Chocolate Candy	Habitual	0.01	0.003	0.01	186.02963	0.719	185.02235	Up
Chocolate Candy	Habitual	0.01	0.003	<0.001	178.0479	0.765	177.04062	Up
Chocolate Candy	Habitual	0.01	0.003	0.03	674.23792	0.804	673.23064	Up
Chocolate Candy	Habitual	0.01	0.003	0.04	250.01481	1.189	249.00753	Up
Chocolate Candy	Habitual	0.01	0.003	<0.001	152.03355	1.313	151.02627	Up
Chocolate Candy	Habitual	0.01	0.003	0.04	190.04791	1.342	189.04063	Up
Chocolate Candy	Habitual	-0.01	0.002	0.01	118.02677	1.371	117.01949	Down
Cakes, puddings & cookies		0.004	0.001	0.04				Up
Chocolate Candy	Habitual	0.01	0.003	0.02	193.98843	1.448	192.98115	Up
Chocolate Candy	Habitual	0.01	0.003	0.01	220.08473	1.731	219.07745	Up
Chocolate Candy	Habitual	0.01	0.003	0.04	145.07391	1.733	144.06663	Up
Chocolate Candy	Habitual	0.01	0.003	0.005	198.05291	1.873	197.04563	Up

Chocolate Candy	Habitual	0.01	0.003	0.002	366.14273	1.948	365.13545	Up
Chocolate Candy	Habitual	0.01	0.003	0.002	178.03016	1.969	177.02288	Up
Candy & sweets		-0.01	0.004	0.02				Down
Chocolate Candy	Habitual	0.01	0.003	0.01	261.08492	2.046	260.07764	Up
Chocolate Candy	Habitual	0.01	0.003	0.03	328.10195	2.065	327.09467	Up
Chocolate Candy	Short-term	0.01	0.002	0.005	166.04926	2.068	165.04198	Up
	Habitual	0.01	0.003	<0.001				
Cakes, puddings & cookies	Habitual	0.005	0.002	0.04				
Chocolate Candy	Short-term	0.01	0.002	0.04	196.05844	2.103	195.05116	Up
	Habitual	0.01	0.003	<0.001				
Chocolate Candy	Habitual	0.01	0.003	0.03	242.98385	2.147	241.97657	Up
Chocolate Candy	Short-term	0.01	0.002	0.01	220.00379	2.215	218.99651	Up
Chocolate Candy	Short-term	0.01	0.002	0.005	116.04765	2.222	115.04037	Up
	Habitual	0.01	0.003	<0.001				
Chocolate Candy	Habitual	0.01	0.003	0.005	285.09626	2.247	284.08898	Up
Chocolate Candy	Habitual	0.01	0.003	<0.001	311.12305	2.422	310.11577	Up
Chocolate Candy	Habitual	0.01	0.003	0.003	383.10778	2.488	382.1005	Up
Chocolate Candy	Habitual	0.01	0.003	<0.001	232.00406	2.784	230.99678	Up

Chocolate Candy	Habitual	0.01	0.003	0.01	412.13438	2.976	411.1271	Up
Chocolate Candy	Habitual	0.01	0.003	0.003	337.07991	3.25	336.07263	Up
Chocolate Candy	Habitual	0.01	0.003	<0.001	351.09545	3.412	350.08817	Up
Chocolate Candy	Short-term	0.01	0.002	0.02	191.05835	3.519	190.05107	Up
	Habitual	0.02	0.003	<0.001				
Chocolate Candy	Short-term	0.01	0.002	0.03	214.08434	3.873	213.07706	Up
Chocolate Candy	Habitual	0.01	0.003	0.002	244.08483	3.96	243.07755	Up
Chocolate Candy	Habitual	0.01	0.003	0.003	244.08483	4.297	243.07755	Up
Chocolate Candy	Habitual	0.02	0.003	<0.001	212.06866	4.104	211.06138	Up
Chocolate Candy	Habitual	0.02	0.003	<0.001	426.15287	4.275	425.14559	Up
Chocolate Candy	Habitual	0.02	0.003	<0.001	460.15831	4.456	459.15103	Up
Candy & sweets		-0.02	0.005	0.004				Down
Chocolate Candy	Habitual	0.02	0.003	<0.001	440.16845	4.612	439.16117	Up
Chocolate Candy	Habitual	0.01	0.003	0.05	542.27294	5.094	541.26566	Up
Chocolate Candy	Habitual	0.01	0.003	0.002	428.20502	5.323	427.19774	Up
Chocolate Candy	Habitual	0.02	0.003	<0.001	444.16339	5.721	443.15611	Up

Chocolate Candy	Habitual	-0.01	0.003	0.01	251.0795	3.357	250.07222	Down
Cakes, puddings & cookies	Habitual	-0.004	0.002	0.04	331.03626	2.79	330.02898	Down

[1]Coefficient are on the log and z scale and adjusted for age, sex, country, batch, BMI z-score and energy intake.
[2]p-value adjusted for false discovery rate (FDR).

Supplementary Table 5 Overview of the features measured in positive ionization mode without annotation associated with short-term dietary intake in IDEFICS/I.Family- results from the sensitivity analysis.

Food Group	Coefficient[1]	SE	p-value[2]	Mass	Retention time	m/z	Regulation
Chocolate Candy	0.01	0.003	<0.001	166.04917[3]	1.896	167.05645	Up
Chocolate Candy	0.02	0.003	<0.001	212.11633	2.667	213.12361	Up
Candy & sweets	0.01	0.005	0.04				
Chocolate Candy	0.01	0.003	0.004	216.11123	1.959	217.11851	Up
Chocolate Candy	0.01	0.003	<0.001	184.08481	1.607	185.09209	Up
Candy & sweets	0.01	0.005	0.04				
Chocolate Candy	0.01	0.003	<0.001	182.04404[3]	1.795	183.05132	Up
Chocolate Candy	0.01	0.003	0.002	143.0585	1.887	144.06578	Up
Candy & sweets	0.02	0.004	0.005	170.10564[4]	2.729	171.11292	Up
Candy & sweets	0.01	0.005	0.03	152.1203	4.71	153.12758	Up
Candy & sweets	0.01	0.005	0.02	328.15222	4.711	329.1595	Up

Candy & sweets	0.01	0.005	0.04	346.16281	4.704	347.17009	Up
Candy & sweets	0.01	0.005	0.03	368.14455	4.71	369.15183	Up
Candy & sweets	0.01	0.005	0.05	310.14162	4.706	311.1489	Up
Candy & sweets	0.02	0.005	0.01	289.12734	1.887	290.13462	Up
Candy & sweets	0.01	0.005	0.03	169.03764	2.222	170.04492	Up
Candy & sweets	-0.01	0.005	0.04	273.19397	3.893	274.20125	Down
Candy & sweets	-0.01	0.005	0.04	287.17315	2.617	288.18043	Down
Candy & sweets	0.01	0.005	0.04	218.04042	0.672	219.0477	Up
Candy & sweets	-0.02	0.004	0.002	327.24097	5.252	328.24825	Down
Candy & sweets	-0.02	0.005	0.002	303.2046	3.705	304.21188	Down
Candy & sweets	-0.01	0.004	0.01	261.15764	1.687	262.16492	Down
Candy & sweets	-0.01	0.004	0.01	243.14718[3]	2.54	244.15446	Down
Candy & sweets	-0.01	0.005	0.03	245.16283	2.678	246.17011	Down
Candy & sweets	-0.02	0.005	<0.001	313.22533[3]	4.951	314.23261	Down

Candy & sweets	-0.02	0.004	<0.001	283.17839[3]	3.549	284.18567	Down
Candy & sweets	-0.02	0.005	0.005	343.23589	3.979	344.24317	Down
Candy & sweets	-0.02	0.004	0.002	341.22022	3.698	342.2275	Down
Candy & sweets	-0.02	0.004	0.005	299.17342	2.95	300.1807	Down
Candy & sweets	-0.01	0.005	0.03	301.18886	3.231	302.19614	Down

[1]Coefficient are on the log and z scale and adjusted for age, sex, country, batch, BMI z-score and energy intake.
[2]p-value adjusted for false discovery rate (FDR).
[3]Has been selected in the main analysis for short-term intake.
[4]In main analysis associated with short-term “chocolate candy” intake.

Supplementary Table 6 Overview of the features measured in negative ionization mode without annotation associated with short-term dietary intake in IDEFICS/I.Family - results from the sensitivity analysis.

Food Group	Coefficient[1]	SE	p-value[2]	Mass	Retention time	m/z	Regulation
Chocolate Candy	0.01	0.003	<0.001	166.04926[3]	2.068	165.04198	Up
Chocolate Candy	0.01	0.003	0.01	116.04765[3]	2.222	115.04037	Up
Chocolate Candy	0.01	0.003	0.03	162.05321	1.638	161.04593	Up
Chocolate Candy	0.01	0.003	0.02	440.08711	0.747	439.07983	Up
Chocolate Candy	0.01	0.003	0.01	436.23104	5.568	435.22376	Up
Chocolate Candy	0.01	0.003	0.02	225.06349	2.695	224.05621	Up
Chocolate Candy	0.01	0.003	0.01	344.07433	2.25	343.06705	Up
Chocolate Candy	0.01	0.003	0.03	378.09293	0.746	377.08565	Up
Candy & sweets	0.01	0.005	0.04				
Chocolate Candy	0.01	0.003	0.01	234.01961	2.118	233.01233	Up
Chocolate Candy	0.01	0.003	0.01	232.00406[4]	2.784	230.99678	Up

Chocolate Candy	0.01	0.003	0.02	344.07433	2.454	343.06705	Up
Chocolate Candy	0.01	0.003	0.02	233.98337	2.255	232.97609	Up
Chocolate Candy	0.01	0.003	0.03	214.08434[3]	3.873	213.07706	Up
Chocolate Candy	0.01	0.003	0.03	426.18915	4.607	425.18187	Up
Chocolate Candy	0.01	0.003	0.03	120.04246	0.842	119.03518	Up
Chocolate Candy	0.01	0.003	0.03	191.05835[3]	3.519	190.05107	Up
Candy & sweets	0.01	0.005	0.04	376.13693	3.134	375.12965	Up
Candy & sweets	0.01	0.005	0.04	169.03793	2.25	168.03065	Up
Candy & sweets	0.01	0.005	0.04	196.05844[5]	2.103	195.05116	Up
Candy & sweets	-0.02	0.005	0.01	174.08944	2.215	173.08216	Down
Candy & sweets	-0.02	0.005	0.01	100.05271	2.8	99.04543	Down
Candy & sweets	0.01	0.005	0.04	289.12758	1.915	288.1203	Up
Candy & sweets	-0.02	0.005	0.01	206.04278	1.753	205.0355	Down
Candy & sweets	-0.02	0.005	0.03	346.1629	5.572	345.15562	Down

Cakes, puddings & cookies	0.003	0.001	0.02	182.04389	1.98	181.03661	Up
Cakes, puddings & cookies	-0.003	0.001	0.05	172.04882	1.288	171.04154	Down
Jelly candy	0.01	0.006	0.04	374.12143	3.886	373.11415	Up
Jelly candy	0.01	0.006	0.04	346.16276	3.846	345.15548	Up
Jelly candy	-0.003	0.001	0.02	243.18361	5.519	242.17633	Down
Jelly candy	-0.01	0.005	0.04	172.04882	1.288	171.04154	Down
Jelly candy	-0.02	0.005	0.01	180.05365	2.808	179.04637	Down
Jelly candy	-0.02	0.006	0.01	346.1629	5.572	345.15562	Down
Jelly candy	-0.02	0.006	0.01	309.10603	0.732	308.09875	Down
Jelly candy	-0.03	0.005	<0.001	100.05271	2.8	99.04543	Down
Chocolate & nut spread	0.01	0.003	0.04	378.09293[6]	0.746	377.08565	Up
Chocolate & nut spread	0.01	0.003	0.04	388.12173[6]	0.759	387.11445	Up

[1]Coefficient are on the log and z scale and adjusted for age, sex, country, batch, BMI z-score and energy intake.
[2]p-value adjusted for false discovery rate (FDR).
[3]Has been selected in the main analysis for short-term intake.
[4]In main analysis associated with habitual “chocolate candy” intake.
[5]In main analysis associated with short-term “chocolate candy” intake.
[6]Features potentially belong to the same metabolite.

Supplementary Table 7 Overview of the repeated double cross-validation with unbiased variable selection (MUVR) test runs on the habitual dietary intake food groups from IDEFICS/I.Family.

Food Group	Feature analysis mode	Method	Prediction Fitness Q^2
Chocolate candy	Negative	RF	0.07
		PLS	0.08
	Positive	RF	0.07
		PLS	0.04
Savory & fatty snacks	Negative	RF	-0.00
		PLS	-0.01
	Positive	RF	0.00
		PLS	-0.01
Candy & sweets	Negative	RF	0.01
		PLS	0.04
	Positive	RF	0.01
		PLS	0.03
Crisps	Negative	RF	0.0
		PLS	0.01
	Positive	RF	-0.05
		PLS	0.01
Cakes, puddings & cookies	Negative	RF	0.05
		PLS	0.02
	Positive	RF	0.03
		PLS	0.02
Ice cream	Negative	RF	0.05
		PLS	0.05
	Positive	RF	0.04
		PLS	0.04

Supplementary Table 8 Results of main regression analysis with repeated double cross-validation with unbiased variable selection (MUVR) algorithms on the habitual dietary intake sample from the IDEFICS/I.Family study.

Food Group	Mode	Algorithm	Original variables	Minimal optimal[2]	Prediction fitness Q^2	*p* for permutation[3]
Chocolate candy	Negative	PLS	316	51	0.082	<0.001
	Positive	RF	383	12	0.081	<0.001
Savoury & fatty snacks	Negative	-[1]	309			
	Positive	-[1]	380			
Candy & sweets	Negative	PLS	311	49	0.047	0.12
	Positive	PLS	390	37	0.043	0.07
Crisps	Negative	PLS	313	25	0.021	-[4]
	Positive	PLS	385	44	0.019	<0.001
Cakes, puddings & cookies	Negative	RF	307	19	0.058	0.01
	Positive	RF	388	18	0.046	<0.001
Ice cream	Negative	PLS	310	49	0.052	-[4]
	Positive	RF	381	11	0.052	

[1]After test-run removed from main analysis.

[2]Number of variables selected as minimal-optimal using MUVR 'min' model.

[3]The permutation p-value of actual model performance vs random permutation distribution (number of permuted models); n=100 permutations.

[4]No features were selected in the linear mixed model (final selection).

Supplementary Table 9 Individual characteristics of the DONALD replication sample.

	**DONALD baseline**<br><br>**N= 297**	**DONALD follow-up**<br><br>**N= 270**
**Median (range)**		
Age	7.0 (3.0 - 10.0)	8.0 (3.9 - 10.3)
BMI z-score[1]	0.0 (-2.3 - 2.7)	0.1 (-2.3 - 2.8)
BMI, kg/m^2	15.8 (12.8 - 23.9)	16.2 (12.9 - 25.9)
Energy intake, kcal/day	1,527.3 (822.8 - 2,801.1)	1,635.2 (687.0 - 2,683.7)
Chocolate intake, grams/day	9.3 (0.0 - 84.7)	9.8 (0.0 - 82.7)
**n (%)**		
Female	157 (53%)	139 (51%)

[1]According to Schaffrath Rosario et al. [1]

1. Schaffrath Rosario, A., et al., *Body mass index percentiles for children and adolescents in Germany based on a nationally representative sample (KiGGS 2003–2006).* European journal of clinical nutrition, 2010. **64**(4): p. 341-349.
